# Bilateral secondary testicular, epididymal and spermatic cords carcinoma of prostatic origin: a case report and review of the literature

**DOI:** 10.1186/s13256-021-02807-4

**Published:** 2021-05-01

**Authors:** Ifeoluwa S. Olorunsola, Amarachukwu C. Etonyeaku, Blessing O. Lekwa, Olusegun S. Ojo

**Affiliations:** 1 Department of Morbid Anatomy and Forensic Medicine, Obafemi Awolowo University Teaching Hospitals Complex (OAUTHC), Ile-Ife, Osun Nigeria; 2 Department of Surgery, Obafemi Awolowo University Teaching Hospitals Complex, Ile-Ife, Osun Nigeria; 3 Mishmael Hospitals and Clinics, Akure, Ondo Nigeria

**Keywords:** Cancer, Prostate, Testes, Metastasis, Orchidectomy

## Abstract

**Background:**

Prostatic carcinoma is emerging as the most common male malignancy in Nigeria and the second most common male cancer worldwide. Patients often present with locally advances stages, and common sites of metastasis are the spine, pelvis, chest, and long bones. Metastases to the testes and spermatic cords are reputed to be rare and may be indicative of a worse outcome, when they occur. We recently encountered a clinical case of bilateral testicular, epididymal and spermatic cords prostatic cancer metastases.

**Case presentation:**

A 71-year-old Nigerian man, who presented at our hospital with 1-month-old complaints of inability to walk together with low back and bilateral thigh pains. This presentation had been preceded by a 5-month history of lower urinary tract symptoms. On examination, the prostate was hard and nodular as were the left testis and spermatic cord. On histological assessment of a needle biopsy, prostatic adenocarcinoma (Gleason score 5 + 5 = 10) was diagnosed. A subsequent therapeutic bilateral total orchidectomy specimen was found to contain metastatic prostatic carcinoma deposits, in the testes, epididymides, and spermatic cords. Although our patient is currently doing well postoperatively on zoledronic acid, ketoconazole, bicalutamide, and tamsulosin, he is being re-evaluated periodically for any feature of recurrence.

**Conclusion:**

Since it has implications for eventual outcome, every clinically suspicious therapeutic orchidectomy specimen should be subjected to a detailed histopathological examination in order to exclude secondaries from the primary prostatic malignancy.

## Background

Carcinoma of the prostate gland is one of the leading cancers in men worldwide, and the incidence and mortality still appear to be on the rise [1]. According to the World Health Organization (WHO), there were about 1.3 million new cases and 359,000 associated deaths in 2018, making prostate cancer the second most frequent cancer and the fifth most common cause of cancer-related deaths worldwide [2]. In Nigeria and other sub-Saharan African, prostate cancer occupies the first position both in incidence and cancer-related mortality [3].

Patients with prostate cancer may present with an array of obstructive and irritative lower urinary tract symptoms as well as weight loss and hematuria [4]. Metastasis is a common occurrence in advanced disease and the usual metastatic sites include the bones, lymph nodes, lungs, and liver [4]. In an autopsy series of 1589 patients with the disease, Bubendorf* et al*. reported that 35% had hematogenous metastatic spread to different organs which included bones (90%), the lungs (46%), the liver (25 %), the pleurae (21%), and the adrenal glands (13%) [5]. Testicular and epididymal metastases are very rare and are reported to have an incidence of between 0.18% and 0.5% [5, 6]. They are usually unilateral, although bilateral cases have been reported [7]. Findings of testicular metastases are mostly incidental on postmortem examination or orchidectomy either as part of the hormonal therapy or for other indications [4]. However, testicular metastases may present as testicular lumps, sometimes [8].

There have been many published reports on testicular metastasis from carcinoma of the prostate. However, to date, to the best of our knowledge, there has been only one case report in the literature on bilateral testicular, epididymal and spermatic cords metastases from carcinoma of the prostate by Johansson and Lannes in 1983 [9]. We therefore present the case of an elderly man who we found to have had bilateral testicular, epididymal and spermatic cords metastases from prostatic adenocarcinoma.

## Case presentation

A 71-year-old Nigerian man presented in our hospital with 1-month-old complaints of inability to walk and low back and bilateral thigh pains. This presentation had been preceded by a 5-months history of lower urinary tract symptoms which were both irritative and obstructive. The pain was a dull ache in nature and had no relieving factors but was aggravated by any attempt to ambulate. There was also a poor appetite and a progressive weight loss in addition. He had no other pre-existing diseases. He was first managed at another hospital where he was fitted with urethral catheter for the relief a sudden inability to void urine.

**Fig. 1:**
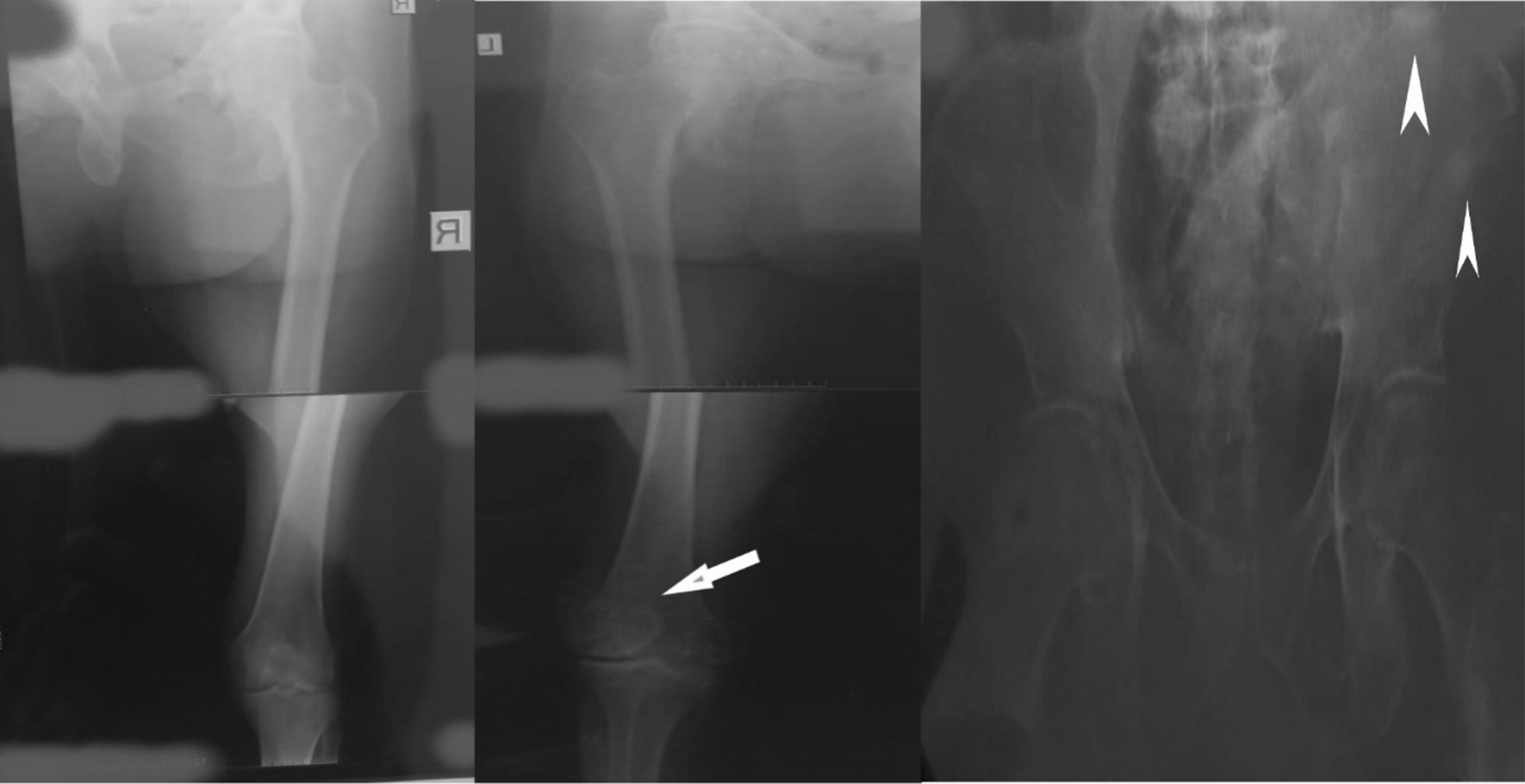
X-ray of the pelvis and right and left femur showing sclerotic lesions on the left iliac bone (arrowheads) and left lower femur (arrow)

At presentation, he was confined to a wheelchair and was in painful distress. He was pale, not jaundiced, mildly dehydrated but had no leg swelling or enlarged lymph node anywhere on the body. He had a urinary catheter in place and this was draining straw-colored urine. His vital signs were normal. The left lower limb was relatively smaller and showed a foot-drop deformity. There was tenderness over the L4–5 vertebrae, as well as in the hip and knees joints during passive movement. The straight leg-raising sign was positive. The left testis and spermatic cord were hard and nodular. On a digital rectal examination, the prostate gland was found to be enlarged, hard, and nodular, but the rectal mucosa was mobile over it. Blood tests revealed a hematocrit of 21%, a prostate-specific antigen (PSA) blood level of 6.8 ng/ml, and normal electrolytes, urea, and creatinine values. The liver function test showed marginally elevated liver enzymes (aspartate and alanine transaminase) and bilirubin. Blood levels of acid phosphatase were also marginally elevated (41 U/L; normal 9–35 U/L). A pelvic ultrasound scan revealed a 57-g enlarged prostate gland having a heterogenous echotexture. An X-ray examination of the femurs showed rounded sclerotic lesions in the distal left femur and left iliac bone (Fig. 1). Abdominopelvic computerized tomography (CT) scan could not be done because of financial constraints. He was resuscitated with intravenous fluids, blood transfusion, and an antibiotic regimen (based on urine microbiological studies). He was also given analgesics and was anticoagulated using enoxaparin. A prostate biopsy obtained through a digitally guided transrectal procedure revealed an infiltrating adenocarcinoma of the prostate (Gleason score 5 + 5 = 10), on histopathological examination. He subsequently had a therapeutic bilateral total orchidectomy carried out; the right testis and epididymis was removed via a trans-scrotal incision, while the left testis and spermatic cord was excised via a groin incision. He was discharged on the second postoperative day.

## Pathology examination

**Fig. 2:**
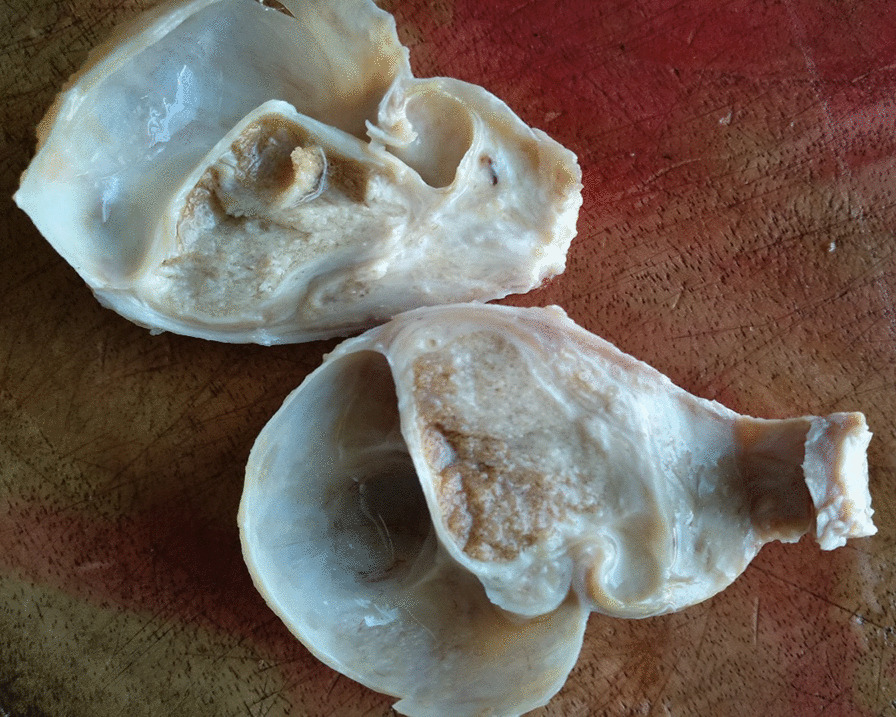
Macroscopic picture of the left testis showing shrunken testicular tissue with grayish white areas as well as a hydrocele

Macroscopic examination of the orchidectomy specimens showed both testes with their respective spermatic cords. The right and left testes measured 4.5 × 3.0 × 2.5 cm and 5.5 × 5.0 × 3.7 cm, respectively. Each spermatic cord measured 5.0 cm in length. Cut surfaces of the right testis were unremarkable while those of the left testis showed a relatively smaller testicular tissue which had been pushed up by a hydrocele that was situated at the inferior pole of the testis. The left testis displayed a golden-yellow appearance which was mottled by a grayish-white discoloration in areas (Fig. 2)

**Fig. 3:**
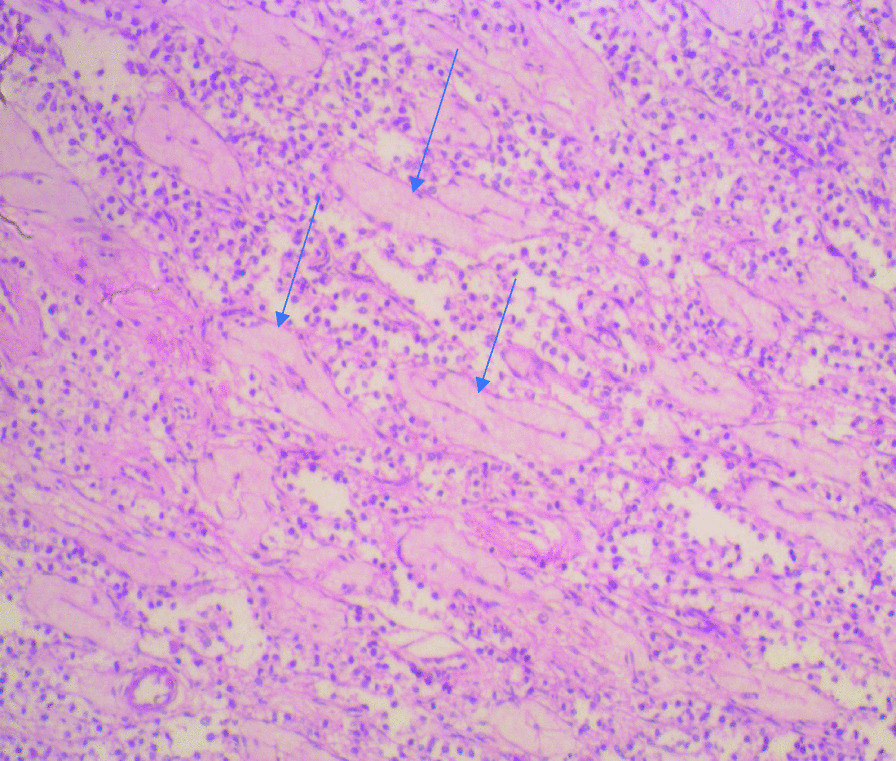
Histological sections showing atrophic seminiferous tubules (blue arrows) and infiltration of the interstitium by malignant epithelial cells occurring in sheets and singly. (Hematoxylin and eosin ×100)

**Fig. 4:**
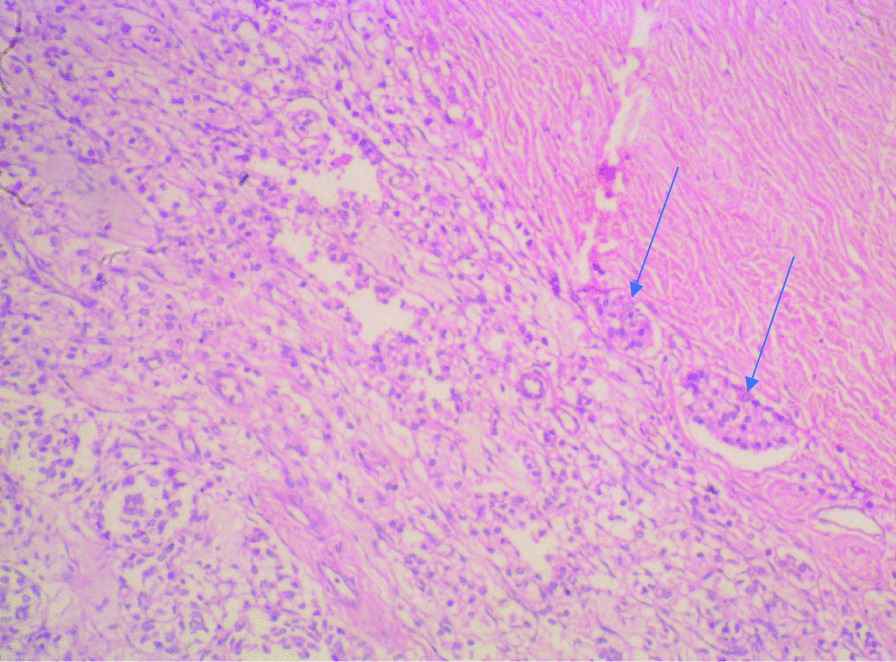
Histological section showing interstitial infiltration and lymphovascular invasion of the malignant cells (blue arrow). (Hematoxylin and eosin ×100)

**Fig. 5:**
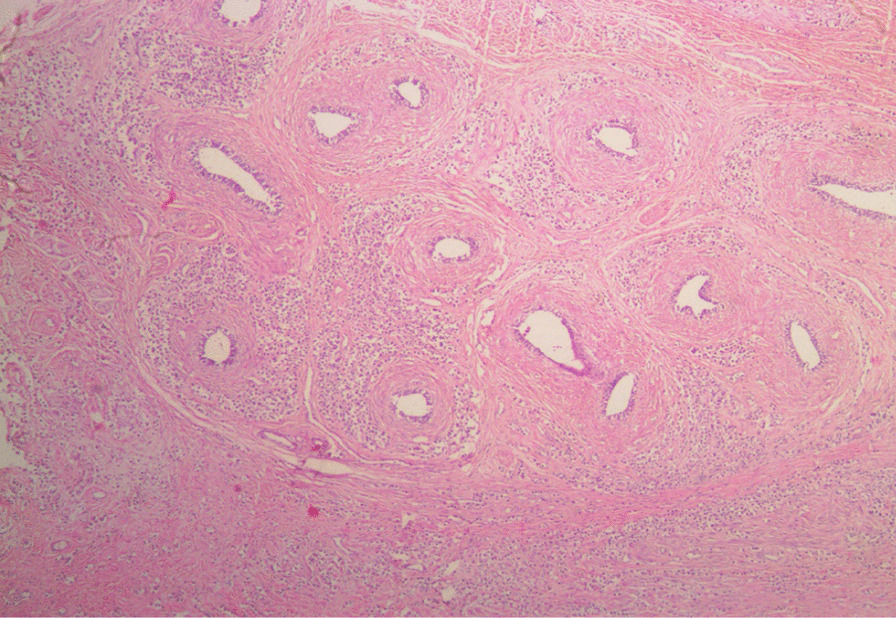
Infiltration of the epididymis by the malignant epithelial cells. (Hematoxylin and eosin ×40)

**Fig. 6:**
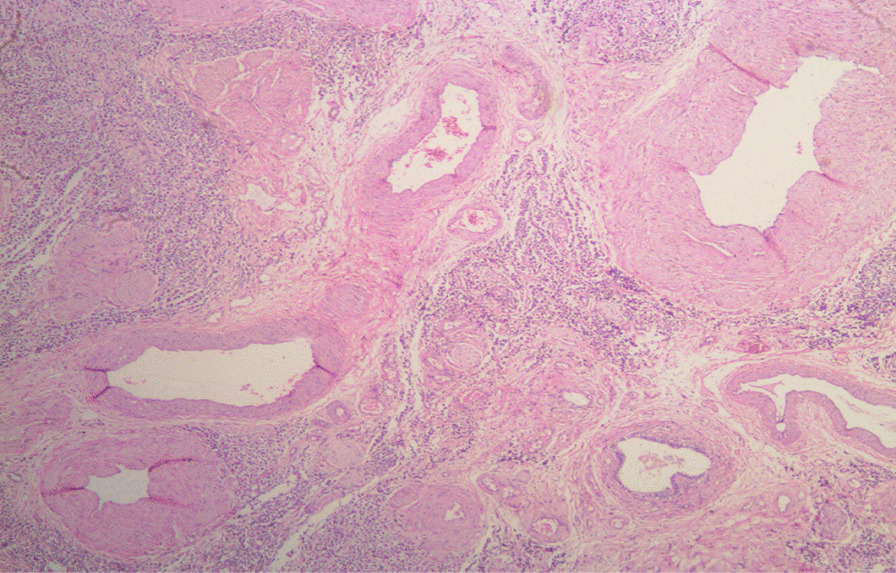
Infiltration of the spermatic cord by the malignant epithelial cells. (Hematoxylin and eosin ×40)

Histological sections from the right and left testes were similar but more remarkable on the left testicle. They showed seminiferous tubules displaying varying degrees of atrophy. There were clusters, cords, and singly-occurring malignant epithelial cells that were infiltrating the interstitium and compressing the seminiferous tubules (Fig. 3). The infiltrating malignant cells were small-sized, uniform, and with hyperchromatic nuclei and scanty cytoplasms. There was a lymphovascular invasion by the malignant cells in areas (Fig. 4). The epididymides and spermatic cords were also extensively infiltrated by the malignant cells (Figs. 5, 6).

**Fig. 7:**
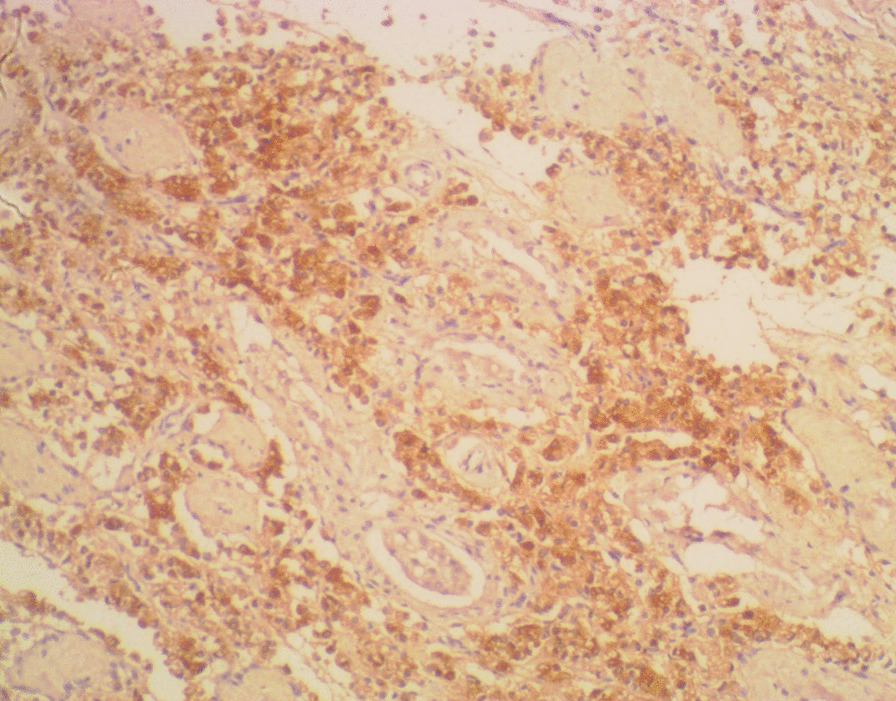
Section showing diffuse and strong prostate-specific antigen immunohistochemistry. (3,3′-Diaminobenzidine ×100)

Immunohistochemistry showed strong cytoplasmic staining for the PSA (Fig. 7). A diagnosis of metastatic carcinoma of the testes, epididymides and spermatic cords from the prostatic adenocarcinoma was made.

He was subsequently placed on yearly zoledronic acid 5 mg given over 15 min, ketoconazole 200 mg twice daily for the bone metastasis, bicalutamide 50 mg daily to ensure maximal androgen blockade, and tamsulosin 0.4 mg daily to improve urine flow. His urethral catheter was discontinued 4 weeks post surgery, without any untoward event. A second post-surgery review was carried out 6 months after and this showed that he ambulates well, although with the aid of a walking stick, while the low back pain and lower limb pain were much reduced. Further follow-up is scheduled.

## Discussion

Testicular tumors may be primary or secondary; primary tumors are more common, and tend to occur in younger male individuals [10]. Metastatic disease of the testes arising from solid tumors are uncommon [11, 12], and reasons adduced for this relatively low propensity for metastatic deposits in the testes have hinged on the existence of a putative blood–testis barrier, and the relatively lower scrotal temperature than the body’s physiological temperature, a condition that might not favor the growth of non-native cells there [4, 12].

Prostate carcinomas are common globally. According to one study, they are the most common male malignancy in Nigeria [3]. Although no appropriate cancer registry data is available, there is a suspicion that there may be a steady rise in the incidence and mortality in Nigeria [3]. In addition, Nigerian patients tend to present in advanced stages of the disease [13].

Worldwide, common metastatic sites of the disease are the bones, lymph nodes, lungs, and liver [5]. Metastatic lesion to the testis and/or spermatic cords is very rare [5, 6], and may be indicative of a worse outcome, when they occur [8, .14]. Olapade-Olaopa * et al.* [11] reported an incidence of 1.4% for testicular metastasis from prostate cancer in a cohort of 142 patients with confirmed prostatic cancer who underwent bilateral orchidectomy.

Patients with testicular metastasis from prostatic adenocarcinoma would have had a background history of lower urinary tract symptoms: predominantly irritative (frequency, nocturia, dysuria, urgency, urge incontinence) compared with obstructive features (hesitancy, staining) [1, 15].

The majority of cases of prostate carcinoma with testicular metastasis have advanced disease with metastasis to other parts of the body, as our patient did [7, 8]. However, only few cases of isolated testicular metastasis have been reported in the literature [6, 16, 17]. While many of these cases are usually incidental findings, few may present with a lump or a nodule, like our patient did [8]. They are also usually associated with high Gleason histological score [16, 17]. Our patient had a Gleason score of 5 + 5 = 10.

The mechanism of spread to the testes has been the subject of debate for decades [17]. The putative routes of spread include retrograde venous extension through the spermatic vein, arterial embolism, retrograde lymphatic extension through the para-aortic lymph nodes, intraductally, through the vas deferens lumen, transperitoneally through a patent vaginal process, and direct invasion [4, 18]. We surmise that the most probable route of spread in our patient is through a retrograde venous extension because of the associated multiple bony metastases.

Morphologically, testicular metastases are usually similar to the primary prostate tumor and may, as such, occur as glandular, cribriform, or single cells invading the interstitium while sparing the seminiferous tubules [15]. However, they may also present with a nodular or destructive pattern, affecting some of the seminiferous tubules or causing them to be severely atrophic, as seen in our case. Microscopy may also show a more aggressive phenotype or a combination of other patterns different from the original histological picture in the primary prostatic tissue [15].

The prognostic implications of a testicular metastasis from prostate cancer are still controversial. Some studies report a 100% mortality within 1 year of diagnosis [8, .14, 19], while others report disease-free intervals, and undetectable serum PSA levels, beyond 5 years post orchidectomy [6, 18].

## Conclusion

This case emphasizes the importance of a thorough histopathological examination of clinically suspicious therapeutic orchidectomy specimens for possible metastases from the primary prostatic carcinoma. This would assist in formulating a more appropriate prognosis in cases where they exist.

## Data Availability

Not applicable.
